# Spontaneous upper limb monoplegia secondary to probable cerebral amyloid angiopathy

**DOI:** 10.1186/1865-1380-5-1

**Published:** 2012-01-03

**Authors:** Ahmed-Ramadan Sadek, Nandita K Parmar, Norah-Hager Sadek, Sanjana Jaiganesh, Samer Elkhodair, Thiagarajan Jaiganesh

**Affiliations:** 1 Wessex Neurological Centre, Southampton University Hospitals NHS Trust, Tremona Road, Southampton SO16 6YD, UK; 2 Division of Clinical Neurosciences, School of Medicine, University of Southampton, Tremona Road, Southampton SO16 6YD, UK; 3 Emergency Medicine Department, St. Georges Hospital, Blackshaw Road, Tooting, London, SW17 0QT, UK; 4 James Allen School, Dulwich, London, UK

## Abstract

Cerebral amyloid angiopathy is a clinicopathological disorder characterised by vascular amyloid deposition initially in leptomeningeal and neocortical vessels, and later affecting cortical and subcortical regions. The presence of amyloid within the walls of these vessels leads to a propensity for primary intracerebral haemorrhage. We report the unusual case of a 77-year-old female who presented to our emergency department with sudden onset isolated hypoaesthesia and right upper limb monoplegia. A CT scan demonstrated a peripheral acute haematoma involving the left perirolandic cortices. Subsequent magnetic resonance imaging demonstrated previous superficial haemorrhagic events. One week following discharge the patient re-attended with multiple short-lived episodes of aphasia and jerking of the right upper limb. Further imaging demonstrated oedematous changes around the previous haemorrhagic insult. Cerebral amyloid angiopathy is an overlooked cause of intracerebral haemorrhage; the isolated nature of the neurological deficit in this case illustrates the many guises in which it can present.

## Introduction

Cerebral amyloid angiopathy is an important yet often unrecognised cause of primary intracerebral haemorrhage (PICH). Typically amyloid β-protein (Aβ) is deposited within the walls of cortical arteries, veins, capillaries and leptomeningeal vessels [1]. Deposition of Aβ within these structures can lead to infarction and haemorrhage [2,3]. In the absence of definitive histological examination the condition cannot be diagnosed, and cases are termed "probable" or "possible" on the basis of imaging studies. Often the diagnosis is overlooked as a causative agent of PICH despite evidence that suggests up to 40% of elderly brains contain cerebrovascular amyloid [4]. Indeed post-mortem evidence suggests that up to 10% of all PICHs are attributable to cerebral amyloid angiopathy (CAA) [4]. Herein we described, to our knowledge, the unique case of a patient who presented with isolated right upper limb weakness secondary to probable cerebral amyloid angiopathy.

## Case report

**Figure 1:**
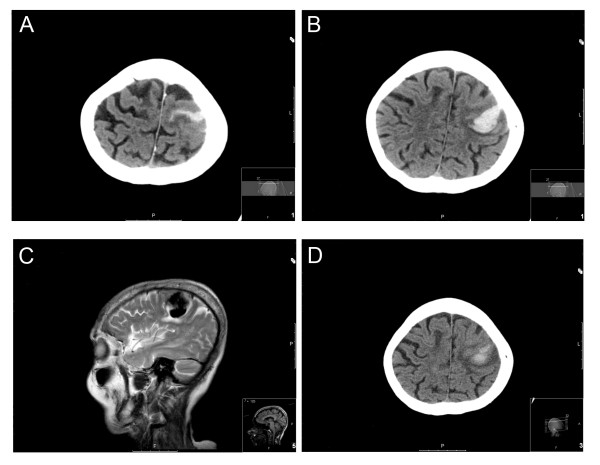
**Localisation of haemorrhagic event**. **(a**, **b) **CT radiographic imaging demonstrating a peripheral acute haematoma involving the left periolandic cortices extending over the left lateral cerebral convexity. **(c) **T2-weighted MRI demonstrating peripherally located haematoma within the left parietal lobe with surrounding oedema and mild compression of the precentral gyrus. **(d) **CT radiographic imaging 7 days later demonstrating maturing left precentral haematoma.

A 77-year-old female presented to our emergency department with a history of sudden right upper limb weakness and altered sensation. The patient was previously fit and well with an unremarkable medical history. On examination, she was apyrexial, normotensive and normoglycaemic with a Glasgow Coma Scale of 15/15. Neurological examination revealed right upper limb hypotonia and power of 0/5 in all hand and wrist muscle groups, 2 over 5 power in biceps and triceps, and 3/5 power in the shoulder girdle. Hypoaesthesia was noted throughout the right upper limb. The remainder of the neurological examination did not reveal any other deficits. Baseline blood investigations were normal. An urgent CT brain was performed and demonstrated a peripheral acute haematoma involving the left perirolandic cortices with extension over the left lateral cerebral convexity (Figure 1a,b). Blood was also noted tracking within the left central sulcus on a background of modest cerebral small vessel disease and generalised cerebral volume loss. Brain MRI with diffusion, and T2 gradient echo demonstrated a haematoma located peripherally within the left parietal lobe with surrounding oedema (Figure 1c) and mild compression of the precentral gyrus in the absence of midline shift. Additionally a mature region of haemorrhage was noted on the right precentral sulcus with an area of superficial cortical scarring and haemosiderin deposition over the surface of the right cerebral hemisphere. Mild ischaemic change was noted throughout the cerebral white matter in the absence of infarcts within the basal ganglia, brainstem or cerebellum. Cerebral venography was not performed, but inspection of the sinuses was not suggestive of a recent thrombosis. Collectively the imaging studies were indicative of an acute parenchymal event with evidence of previous superficial bleeds. Following little improvement in right arm function, after neuro-rehabilitation, the patient was discharged to her usual residence. One week following discharge the patient re-attended our department with multiple 5-min episodes of loss of speech and jerking movement of the right arm. Subsequent CT brain scan and MRI did not demonstrate either repeat haemorrhagic or new ischaemic events. The focal motor seizures were attributed to oedematous changes surrounding the previous haemorrhagic event (Figure 1d). The patient was started on anti-epileptic medication with no further seizure activity prior to discharge.

## Discussion

**Figure 2:**
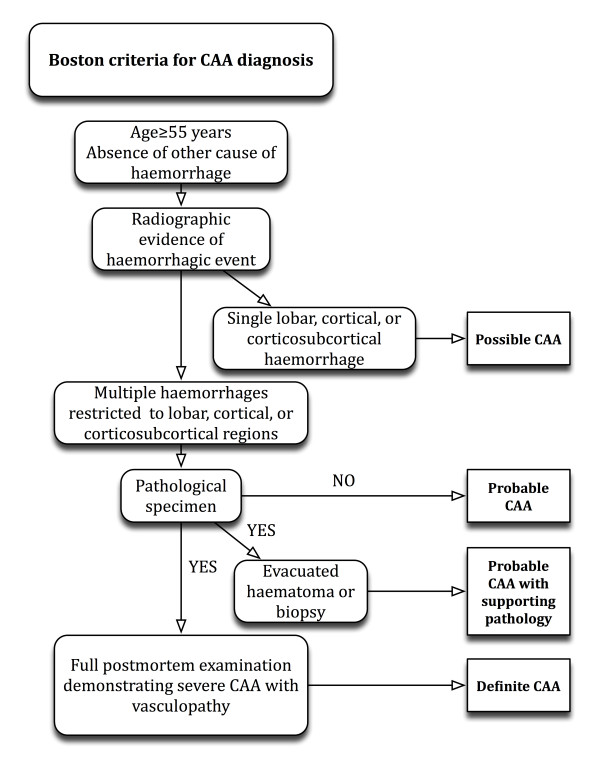
**Boston criteria for cerebral amyloid angiopathy diagnosis**.

Cerebral vascular abnormalities secondary to Aβ deposition were first described over 70 years ago [5]. Cerebral amyloid angiopathy is not an uncommon pathological finding, especially in the aged brain and in those with Alzheimer's dementia [4]. The diagnosis of PICH secondary to CAA is often overlooked, as it is dependent on histological examination of cerebral tissue. In the absence of definitive histopathological examination the diagnosis of PICH secondary to CAA is made on the basis of radiological findings and is termed "probable" or "possible". Probable cases of CAA on radiological examination have evidence of multiple lobar haemorrhagic events, whilst possible diagnoses only possess evidence of a single event (see Figure 2) [6]. The condition is rarely observed in those under 55 years of age [7], and up to 36% of those aged between 60-97 years of age possess varying degrees of CAA on post-mortem examination [8].

Despite the persistent decline in haemorrhagic stroke mortality in those under 74 years of age [9], it is increasingly apparent that those greater than 75 years of age may have an increasing incidence of PICH, which may be attributable to CAA [10]. With improved management of hypertension, the percentage of PICH attributable to CAA is likely to become more evident. Indeed one study has demonstrated that up to 74% of lobar PICHs are secondary to CAA [6].

The clinical manifestations of CAA are variable; however PICH is the most common presentation. There are no pathognomonic clinical features, with many patients being completely asymptomatic. Headaches, altered conscious level, neurological deficits, seizures and cognitive impairment on a background of dementia are recognised symptoms. Spontaneous haemorrhagic events secondary to CAA can also be asymptomatic and are referred to as "microbleeds" [11]. Due to the predilection of Aβ deposition in superficial cortical and leptomeningeal vessels haemorrhagic events tend to be lobar in location [12]; this is in contrast to hypertensive PICHs, which tend not to be. The size of the haematoma is typically related to the extent of the neurological deficit. Previous reports describe patients who present with unilateral hemiparesis or hemiplegia with preceding headache and altered conscious level [13]. Our case was unusual in that the patient only had monoplegia in her right upper limb, with no preceding symptoms. Radiological investigation demonstrated both new and old cerebrovascular events, consistent with a "probable" diagnosis on the basis of the Boston CAA criteria [12] (see Figure 2).

The management of patients with PICH secondary to probable CAA is no different from PICH as a result of any other aetiology. It has been previously thought that Aβ within the media and adventitia of cortical and leptomeningeal blood vessels may interrupt vasoconstriction during haemostasis following an ICH, making neurosurgical intervention unsafe [14]. A large case series has, more recently, demonstrated that neurosurgical intervention can be safely considered in patients < 75 years without parietal and intraventricular haematoma [15]. No strict guidelines exist addressing secondary prevention of PICH due to CAA; however it would seem prudent to manage high blood pressure and advise a reduction in alcohol consumption [7]. There are presently several ongoing drug development studies addressing strategies targeting the production and clearance of Aβ [16]; however none of these agents have yet been approved for use in CAA. Ultimately, clinicians need to be aware that CAA is an important cause of PICH and need to consider it in the elderly patient who has no other clear aetiological cause for their cerebral haemorrhage.

## Abbreviations

CAA: cerebral angiopathy; CT: computerised tomography; ICH: intracerebral haemorrhage; MRI: magnetic resonance imaging.

## Consent

Written informed consent was obtained from the patient for publication of this case report and any accompanying images. A copy of the written consent is available for review by the Editor-in-Chief of this journal.

## Competing interests

The authors declare that they have no competing interests.

## Authors' contributions

ARS wrote the first draft of the paper and coordinated the review of all the drafts. NKP reviewed and commented on all the drafts of the paper. NHS reviewed and commented on all the drafts of the paper. SJ searched and extracted relevant literature for the article. SE reviewed and commented on all the drafts of the paper. TJ reviewed and commented on all the drafts of the paper. All authors reviewed and commented on all radiographic images.

## Funding and sponsorship

None
